# Logbooks alone are not enough: initial experience with implementing a logbook for medical students in a clinical internship in gynecology and obstetrics

**DOI:** 10.1186/s40001-020-00413-6

**Published:** 2020-05-08

**Authors:** Sebastian M. Jud, Susanne Cupisti, Wolfgang Frobenius, Sigrid Benn, Andrea Winkler, Sophia Antoniadis, Matthias W. Beckmann, Felix Heindl

**Affiliations:** Department of Gynecology, Erlangen University Hospital, Universitätsstrasse 21–23, 91054 Erlangen, Germany

**Keywords:** Teaching, Gynecology, Logbook, Internship

## Abstract

**Background:**

Logbooks are being increasingly widely used as a means of improving medical education and further training. They will in all probability continue to be mandatory in the Practical Year (PJ) in Germany even after the upcoming amendment of the Medical Licensing Regulations (ÄAppO). However, there are different approaches to their design and use, and these are also currently undergoing considerable change. This study for the first time examines and discusses the influence of logbooks on students’ evaluation of a gynecology internship.

**Methods:**

The study was based on a well-established two-part 1-week internship course, with initially unstructured morning classes on wards and duty areas, along with precisely planned afternoon classes with skills training by peer teachers and seminars supervised by duty-exempted physicians. The postgraduate lecturers were prepared for the introduction of the logbook in a special course, and the aim was to optimize morning classes by introducing learning objectives adapted to the respective locations. The effects over 38 weeks of practical training were examined in evaluations by 235 prospectively group-randomized students with and without logbooks (*n* = 166 and *n* = 66, respectively; three datasets were not evaluable).

**Results:**

In the cohort comparison, the logbook group responded significantly more positively toward the internship at the start of the course (*P* = 0.046). In the final evaluation, however, medical supervision during the entire internship was rated significantly more poorly (*P* = 0.007). The logbook cohort also considered that guidance based on learning objectives was significantly worse, as was the extent to which wards and duty areas were prepared for the students (*P* = 0.001 and *P* = 0.029).

**Conclusions:**

Introducing a logbook to optimize clinical teaching in internships may raise expectations that cannot always be met. In addition to adapting the learning objectives to a general framework that is less favorable in comparison with the Practical Year, the least that is required appears to be simultaneous and continuous mentoring of the lecturers, as well as an increase in staffing resources.

## Background

Logbooks are being increasingly widely used as a means of improving medical education and further training. In Germany, their use has already been required since 2013 by the Medical Licensing Regulations (*Ärztliche Approbationsordnung,* ÄAppO) for students in their Practical Year (*Praktisches Jahr,* PJ). The PJ is the last year of study where the students are working under supervision in three different clinical departments (internal medicine, surgery and one of their choice). However, logbooks are also to be included in the currently upcoming amendment of the ÄAppO, and an electronic version of them was even included at the end of 2019 for the first time in regulations for further training [1, 2].

However, widely varying approaches exist to the possible design and usage of logbooks. The current ÄAppO does not provide any detailed specifications on this. A working group of the Medical Faculty Association (*Medizinischer Fakultätentag,* MFT), including representatives of specialist associations, already suggested “basic” logbooks for surgery and internal medicine as long ago as 2012 [3]. These have since been modified with the addition of a new approach to produce a “PJ sample logbook 2.0”, which also includes general medicine. This approach focuses above all on implementing an interdisciplinary set of “entrustable professional activities” (EPAs) [4].

The published data so far available on the effectiveness and acceptability of logbooks are very heterogeneous. However, there is widespread agreement that logbooks can in principle be highly beneficial, since on the one hand they structure clinical and practical teaching activities by providing clearly defined learning objectives and making them testable. On the other hand, they provide students with an established curriculum that they can actively request to be provided [5–8]. A pilot study for the Medical Faculty Association’s approach to EPAs has already been published [9].

To the best of our knowledge, there have been no previous studies of the use of logbooks in internship courses in obstetrics and gynecology in Germany. The present study is intended firstly to address the following question: what influence does the introduction of a logbook have on students’ evaluation of clinical teaching in an internship? This then leads to a discussion of the use of logbooks, particularly in internships that also include the EPA concept. These internships play an important role in medical education as it is the last theoretical and practical training before the Practical Year and it serves as a possibility to present the specialty.

## Materials and methods

### Structure of the Erlangen internship

The Erlangen internship course in gynecology and obstetrics was completely restructured in 2007–2008 (Table 1), in order to meet the current ÄAppO requirements to make training courses more practically relevant and to make grading possible. At the same time, the special sensitiveness of the specialty in relation to preserving patients’ privacy and the scarcity of personnel resources also had to be taken into account. The result was the development of a 1-week internship program with a two-part schedule: in the morning, clinical teaching on the wards, in the delivery room and operating room, as well as in the duty area departments; in the afternoon, practical exercises in the skills laboratory together with peer teachers, following precise working instructions, along with seminars headed by physicians on important clinical pictures seen during the morning’s teaching. The final examination takes the form of a partially formative “mini-OSCE” (objective structured clinical examination). Our mini-OSCE consists of six sections, three practical and three theoretical sections. Due to this reduced amount, we used the name mini-OSCE to demonstrate the difference to a normal OSCE with 12 to 35 different parts. Details on the development of the internship have already been published elsewhere [10].

**Table 1:** Structure of the Erlangen internship (from [10])

	Monday	Tuesday	Wednesday	Thursday	Friday
7.30–12.00 a.m.	Induction (in the library)	–	–	–	–
	Practice	Practice	Practice	Practice	Practice
	On the wards	On the wards	On the wards	On the wards	On the wards
	In surgery	In surgery	In surgery	In surgery	In surgery
	In the outpatient clinics (according to plan)	In the outpatient clinics (according to plan)	In the outpatient clinics (according to plan)	In the outpatient clinics (according to plan)	In the outpatient clinics (according to plan)
12.00–1 p.m.	Lunch break	Lunch break	Lunch break	Lunch break	Lunch break
1.00–2.30 p.m.	Skills training:	Skills training:	Skills training:	Skills training:	Final examination 2.00 p.m.
	Speculum insertion	Speculum insertion	Birth simulator	Maternity record	
	Pap smear	Infection diagnosis with inspection, pH and native preparation	Spontaneous delivery	Calculating and correcting due dates	Preliminary discussion 1.50 p.m.
	Explanation of findings		Placental period	Assessing labor (BP, blood count, urine findings)	
			Atony, Apgar		
	Clinical implications	Clinical implications	CTG basics		
2.30-3.00 p.m.,	Coffee break	Coffee break	Coffee break	Coffee break	
3.00–4.30 p.m.	Seminar:	Seminar:	Seminar:	Seminar:	
	Case review	Case review	Contraception, HPV vaccination, gynecol. screening	Case review	
	Breast carcinoma	Gynecologic carcinoma	End: 3.50 p.m.	Complications in pregnancy	

BP, blood pressure; CTG, cardiotocography

With its new structure, the internship course had been able to substantially improve its position in the faculty’s internal ranking of evaluations and has always held one of the top positions over the years. However, detailed analysis of these evaluations showed that the morning classes were a persistent problem, partly because of the lack of competence-based learning objectives. To remedy this situation, a logbook was established that was intended to take into account the fact that scenarios in everyday clinical practice vary from day to day.

### Logbook in gown-pocket format

In addition to general information for guidance in the hospital, behavior in the operating room, and the sequence of the internship course, the gown-pocket format logbook now also includes information about the morning teaching, in addition to the previously well-communicated learning objectives for the afternoon classes. Each of the four to seven items is structured in accordance with the students’ different places of work (e.g., obstetrics, surgery, ultrasound, endocrinology, breast consultation, wards). The educational goal is to achieve at least three of the learning objectives listed for each location, with corresponding confirmation by the physicians who provide the training.

### Study design

Starting from the summer semester of 2012, a total of 38 internship weeks, each including up to six students, were evaluated for the study. A total of 235 students were included. During the first 11 weeks, morning classes were held without logbooks, as before. Over the following 11 weeks, the aim was to use logbooks consistently during the course. In a separate analysis, the following 16 weeks with logbooks were examined separately to clarify possible learning effects in connection with the use of the logbooks.

**Fig. 1:**
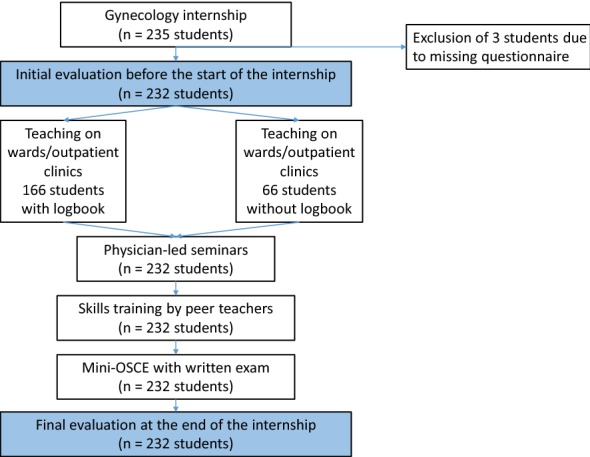
Study design. OSCE, objective structured clinical examination

The students were prospectively assigned to the individual groups on the basis of group randomization, based on their registrations in the online portal at the Dean of Studies Office. The time point at which the logbook was to be introduced was determined independently of that. The postgraduate lecturers were informed about the introduction and use of the logbook in advance, in a one-time further training event. No checking of the logbook entries was initially planned. The other components of the internship did not differ during the period under study (Fig. 1).

At the beginning of the internship, the students taking part completed an anonymized, voluntary questionnaire including six items on their personal details, previous education, and expectations of the internship course (Table 2). At the end of the internship, the students completed an assessment questionnaire including items on their motivation, the structure of the course, the practical training on wards and in the skills training section, as well as on the examination and their overall evaluation (Table 3). With one exception (item 28), a six-point Likert scale was used: 1, strongly agree; 2, mainly agree; 3, somewhat agree; 4, somewhat disagree; 5, disagree; 6, strongly disagree. The six-point scale was chosen in order to avoid any neutral positions.

**Table 2:** Questions included in the initial evaluation questionnaire

No.	Item
1	Age
2	Gender
3	Number of semesters
4	How many clinical internships have you completed in Erlangen?
5	Have you already taken part in a gynecological internship elsewhere?
6	Are your views about the current internship generally more positive or more negative?

**Table 3:** Questions included in the final evaluation questionnaire (with the exception of item 28, possible answers were graded on a Likert scale: 1, “strongly agree” to 6, “strongly disagree”)

No.	Item
7	I was very interested in gynecology even before the internship
8	The internship has increased my interest in gynecology
9	The structure and sequence of the internship were comprehensible
10	The organization and implementation of the course were good
11	There were too many phases in which idle time occurred
12	The supervision by physicians during the whole internship was good
13	I felt I was being integrated into everyday medical work
14	My questions were answered willingly
15	The physicians and nursing staff treated me in a friendly way
16	Things were naturally explained, without questions having to be asked
17	Wards and outpatient clinics were prepared to receive the students
18	The training was based on preset learning objectives
19	I had an opportunity to take part in diagnosis and treatment
20	I received constructive feedback on this from the physicians.
21	I was mainly used for auxiliary activities
22	I repeatedly felt that I was being ignored and pushed away
23	I was able to go into the deeper theoretical content of the specialty in an application-oriented way
24	The theoretical part of the examination was appropriate
25	The practical part of the examination was appropriate
26	The practical part of the exam motivated me to undertake more intensive skills training.
27	Taking part in the internship was worth the time involved
28	I would give the internship the following overall grade (grades on a scale of 1 for “very good” to 6 for “inadequate”) …

### Statistical analysis

The data were stored using Microsoft Excel 2016 (Microsoft Inc., Redmond, Washington, USA), and the analysis was performed using IBM SPSS Statistics for Windows, version 24 (IBM Corporation, Armonk, New York, USA). Fisher’s exact test was used to compare the group characteristics of gender, number of semesters, and previous practical experience. One-way analysis of variance (ANOVA) was used for comparison of means for Likert-scaled items and point values. A *P* value of less than 0.05 was considered statistically significant.

## Results

### Descriptive statistics

A total of 232 datasets were evaluable. The students’ mean age was 25.29 years (SD 2.504) and they were on average in their ninth semester (mean 9.08; SD 0.813). The students had already completed a mean of 4.88 (SD 3.597) internships at Friedrich Alexander University of Erlangen–Nuremberg before attending the course investigated here. Thirty-two students had already taken part in a gynecology internship at another university (13%). Among the students, 41.8% were male and 58.2% female.

### Learning curve

As described above, the original division of the logbook group into two (one early and one late) was intended to identify a possible learning effect in the use of the logbook, both on the part of the medical trainers on the wards and also on the part of the students, who often seek information from their predecessors. However, as the two groups did not differ in their evaluations, they were subsequently analyzed as a single group.

### Cohort comparison

The analysis of the evaluation at the start of the internship showed that the cohorts with logbooks (*n* = 166) and without logbooks (*n* = 66) differed significantly only in their attitude toward the internship. The logbook group had a more positive attitude toward the upcoming course (*P* = 0.046). In contrast, age, gender distribution, and number of semesters were largely similar, as was the level of previous experience with internship placements (in gynecology and other specialties). Most of the students were in their ninth semester and had already completed around five other block internships in Erlangen. The proportion of students who had already taken part in an internship in gynecology elsewhere was just under 14% overall.

### Final evaluation

*Motivation* The final evaluation showed that the gynecology students had a fairly moderate degree of interest in the specialty before the internship course (item 7; mean Likert score in both groups around 3). During the internship, however, it was apparently possible to achieve at least a moderate increase in interest in the specialty in both cohorts studied (item 8, mean Likert score 2.35 in the logbook group, 2.21 without logbooks). However, a significant effect of the introduction of the logbook was not confirmed (*P* = 0.428).

*Structure of the internship* All of the students rated the structure of the internship course as being very good to good (items 9, 10, and 11). Here again, however, the introduction of the logbook evidently did not have any significant influence. Even when asked about “idle” periods in the process, the students were relatively satisfied either with or without logbooks (item 11: mean Likert score in both groups around 4—i.e., too much “idling” tended not to occur). However, the item did not allow any differentiation between the mornings on wards and in duty areas and the afternoon classes with peer teachers and specially duty-exempted physicians.

*General conditions in practical training* Items 12 to 17 were mainly concerned with the general conditions of the practical training provided in the morning classes on the wards, which were to be improved through the introduction of the logbook. At first sight, there was a surprising effect here in that the group with logbooks gave the medical supervision provided during the whole of the practical training course a significantly poorer evaluation than the cohort without logbooks did (item 12, *P* = 0.007). The same also applied to the degree of preparation to receive the students who were experienced in the outpatient clinics and wards (item 17, *P* = 0.029). This aspect is considered in the following Section “Discussion”. Notwithstanding this, it should be noted that students both with and without logbooks all rated the medical supervision as being good overall (mean Likert score under 2), as well as the way the physicians and nursing staff treated students (items 14 and 15). The extent of integration into the physicians’ everyday work that was allowed and of physicians’ willingness to offer spontaneous explanations were rated significantly worse (items 13 and 16: mean Likert score around 2.5) (Table 4).

**Table 4:** General conditions in the practical training

No.	Item	With logbook		Without logbook		*P* value
		Mean	SD	Mean	SD	
12	The supervision by physicians during the whole internship was good	1.88	0.862	1.56	0.712	0.007
13	I felt I was being integrated into everyday medical work	2.49	1.197	2.35	1.057	0.412
14	My questions were answered willingly	1.55	0.867	1.43	0.844	0.326
15	The physicians and nursing staff treated me in a friendly way	1.76	0.908	1.75	0.857	0.900
16	Things were naturally explained, without questions having to be asked	2.57	1.221	2.43	1.137	0.394
17	Wards and outpatient clinics were prepared to receive the students	1.97	1.116	1.63	0.960	0.029

The items were graded on a Likert scale from 1, “strongly agree” to 6, “strongly disagree”

*Practical work on wards and in duty areas* In the question on the extent to which the training course was oriented toward preset learning objectives (item 18), a similar effect to that in items 12 and 17 (see above) was observed: the logbook cohort rated this item significantly worse than the students who did not have learning objectives for the morning sessions (*P* = 0.001). This point is additionally worthy of discussion, as it must be assumed that the cohort without logbooks were basing their good assessment (mean Likert score 1.61) only on the afternoon learning objectives that were available at the time.

The comparatively modest evaluation of the clinical and practical teaching provided in the mornings—which together with negative free-text comments had originally given rise to the introduction of the logbooks—remained almost completely unaffected by the measure, as is shown by the other results of the investigation (items 19–23) (Table 5).

**Table 5:** Evaluation of the practical training on the wards/outpatient clinics

No.	Item	With logbook		Without logbook		*P* value
		Mean	SD	Mean	SD	
18	The training was based on preset learning objectives	2.11	1.127	1.61	0.889	0.001
19	I had an opportunity to take part in diagnosis and treatment	2.70	1.268	2.70	1.247	0.981
20	I received constructive feedback on this from the physicians	2.86	1.320	2.90	1.264	0.841
21	I was mainly used for auxiliary activities	4.26	1.609	4.48	1.491	0.340
22	I repeatedly felt that I was being ignored and pushed away	5.08	2.243	4.96	1.270	0.665
23	I was able to go into the deeper theoretical content of the specialty in an application-oriented way	2.17	1.036	2.06	0.939	0.440

The items were graded on a Likert scale from 1, “strongly agree” to 6, “strongly disagree”

*Examinations* For obvious reasons, the final examination, which was designed and graded as a “mini-OSCE” with theoretical and practical sections, primarily focuses on the knowledge and skills taught in the seminars and in the skills training sessions. It is not possible to cover the learning objectives from the morning teaching, or only in a rudimentary way. Nevertheless, the evaluation showed a significant difference in the appropriateness of the theoretical examination questions between the groups with and without logbooks: the logbook group was more critical on this point.

Other evaluations of the examination did not show any differences between the groups. Unsurprisingly, the testing of skills in the examination motivates students to undertake more intensive training (Table 6).

**Table 6:** Comparison of evaluations of the examination

No.	Item	With logbook		Without logbook		*P* value
		Mean	SD	Mean	SD	
24	The theoretical part of the examination was appropriate	1.64	0.799	1.41	0.575	0.032
25	The practical part of the examination was appropriate	1.48	0.77	1.39	0.547	0.375
26	The practical part of the exam motivated me to undertake more intensive skills training	2.3	1.3	2.27	1.183	0.841

The items were graded on a Likert scale from 1, “strongly agree” to 6, “strongly disagree”

*Overall evaluation of the internship* The overall assessment of the internship was good. There were no significant differences between the two groups. Students both with and without logbooks considered that it was worth the time spent (mean Likert scores 1.69 and 1.7, respectively). The mean grades awarded for the practical training were 1.57 and 1.56 (on a scale of 1 for “very good” to 6 for “inadequate”).

*Free text* (*qualitative evaluation*) The evaluation of free-text comments (*n* = 17; 10 in the group with logbooks, 7 in the group without) was no more conclusive in relation to logbook-relevant practical training on the wards or in the outpatient clinics than the quantitative evaluation of the above-mentioned items. The free-text comments—both negative and positive—merely put the information in verbal form. In all, 10 comments were positive and 7 comments were negative (with no significant difference between the groups). There were no critical comments on the logbooks. There were several complaints about the lack of supervision on the wards and in the outpatient clinics.

## Discussion

In view of the high reputation that logbooks have for providing competence-oriented, practical clinical training with operationalized learning objectives, the results of the present study must appear at first glance to be both surprising and sobering. In an internship in gynecology and obstetrics that has in principle received good evaluations over many years, the introduction of a logbook procedure led to significant deterioration in the course in the eyes of the students, and particularly in connection with the factors that it was actually intended to further improve: supervision by physicians, orientation toward learning objectives, and the extent to which the wards were prepared to receive the students. This is probably one of the reasons why the logbook did not significantly increase the students’ level of interest in the subject, which was initially fairly moderate.

However, the initially surprising results of the investigation are actually explicable on closer examination. Firstly, they are probably the result of a phenomenon often associated with innovations: raising a higher level of expectations, which are then inevitably disappointed to some extent (see item 6). Secondly, it is very likely that the new learning objectives for the clinical and practical morning classes, which were explicitly articulated for the first time, made the students’ otherwise rather vague expectations clearer. This then also made it clear to them that there were apparently also discrepancies between desirable goals in the curriculum (specified learning objectives) and the practical realities of teaching (learning objectives that were actually achievable or achieved) on the wards and in the duty areas.

In contrast, the group without logbooks inevitably only had the already well-structured afternoon classes in mind in relation to the specified learning objectives. For these students, the peer teaching events, which were always dependably held on fixed dates, and the seminars given by duty-exempted medical staff led to the better evaluation described above. Above all, the regularly supervised peer teaching in accordance with explicit working instructions probably played an important role here—not least because the skills taught, as reliably achievable learning objectives, also represented the content that was tested in the final OSCE. The learning objectives for the morning classes listed in the logbook were not capable of meeting all of the criteria mentioned. This is at any rate suggested by the students’ significantly poorer assessment of the overall medical supervision, although it was still regarded as being good.

Other groups have also described similar experience with the use of logbooks in the context of the Practical Year. Busemann et al. reported on a negative evaluation of a 4-month surgery course. Fewer than half of the students on the course had the impression that logbooks improved their training. Major points of criticism were that there was a lack of guidance in practical activities and a lack of adequate feedback. Allocating more time for the physicians involved in teaching was proposed as a consequence. However, the study was not a prospective comparison of two cohorts with and without logbooks, but a retrospective, anonymized survey (response rate 54%) of 70 students who had all used logbooks [5].

Several conclusions can be drawn from our own experience with the introduction of a logbook into clinical teaching, as well as from others’ unfavorable experience with logbooks. Basically—and unsurprisingly—positive results can only be expected if there are sufficient staff on the wards to make it possible to implement and check the specified learning objectives (including qualitative feedback), at least to a substantial extent. Another conclusion is that it is not sufficient to present the new approach at a single meeting to the physicians who are to do the teaching, and that consistent monitoring is necessary. In addition, it seems important to avoid overambitious specifications for the existing learning objectives—i.e., their relevance and feasibility need to be critically reviewed again and again. This requirement is also important during the development of the EPAs mentioned above [9].

Particularly for internships, it should be remembered that the situation of teachers and students in this setting is substantially different from the Practical Year. The instructors on the wards and in duty areas face new students every day, while the students for their part rotate through four to five clinical units within a week, so that they also face not only new staff every day, but also a new environment. This makes teaching considerably more difficult, and it makes the transference (“entrusting”) of complex professional activities almost impossible, so that it will probably have to be mainly reserved for the Practical Year. Not least for this reason, the German Council of Science and Humanities (*Wissenschaftsrat*) has recommended that in the new Medical Licensing Regulations (ÄAppO), internships should be converted into “clinical auditorships … to allow students to experience and acquire a participatory-observational understanding of in-patient and outpatient care processes” [11].

However, the approach that we have taken to skills training, embedded in a clinical context, which has also been well evaluated, can be regarded as a desirable preliminary stage for the type of Practical Year training that is being discussed in connection with EPAs [4]. It therefore appears useful to maintain this approach and focus above all on a critical revision of the logbook’s learning objectives in the morning classes, to intensify the training offered to the physicians acting as instructors, and to look for further opportunities to expand staffing resources, on the one hand, and also to use them sparingly on the other.

The present study is only an initial step toward investigating the effectiveness of using logbooks in internships in gynecology and obstetrics. All that was analyzed was the evaluation of the course by two cohorts of students who completed the internship either with or without logbooks. There was no rigorous checking either of whether the logbooks were properly kept, or of whether the learning objectives selected were actually achieved. Clearer differentiation at some points on the evaluation sheet between the morning and afternoon classes would probably have been helpful.

## Conclusion

Logbooks in internship courses need to meet different requirements from the logbooks used in the Practical Year. The special situation of these students in the clinical setting, in which transference of “entrustable professional activities” (EPAs) is scarcely possible, has to be taken into account when the learning objectives are being formulated. However, more complex forms of skills training embedded in the clinical context and seminars are certainly able to make a valuable contribution to students’ preparation for the Practical Year.

## Data Availability

All data of the questionnaires and characteristics of the participants are available.
